# Variability of CP4 EPSPS expression in genetically engineered soybean (*Glycine ma*x L. Merrill)

**DOI:** 10.1007/s11248-018-0092-z

**Published:** 2018-09-01

**Authors:** Parimala Chinnadurai, Duška Stojšin, Kang Liu, Gregory E. Frierdich, Kevin C. Glenn, Tao Geng, Adam Schapaugh, Keguo Huang, Andrew E. Deffenbaugh, Zi L. Liu, Luis A. Burzio

**Affiliations:** Bayer Crop Sciences, 700 Chesterfield Parkway West, St. Louis, MO 63017 USA

**Keywords:** Soybean, Glyphosate-tolerance, CP4 EPSPS, Expression variation, MOE, GE crops

## Abstract

The expression of the CP4 EPSPS protein in genetically engineered (GE) soybean confers tolerance to the *Roundup*^®^ family of agricultural herbicides. This study evaluated the variability of CP4 EPSPS expression using an enzyme-linked immunosorbent assay in soybean tissues collected across diverse germplasm and 74 different environments in Argentina, Brazil and the USA. Evaluated material included single and combined (stacked) trait products with other GE traits in entries with *cp4 epsps* gene at one or two loci. The highest level of CP4 EPSPS was observed in leaf tissues, intermediate in forage and seed, and lowest in root tissues. Varieties with two loci had approximately twice the level of CP4 EPSPS expression compared to one locus entries. Variable and non-directional level of CP4 EPSPS was observed with other factors like genetic background, trait stacking, growing region or season. The maximum and average CP4 EPSPS expression levels in seed provided large margins of exposure (MOE of approximately 4000 and 11,000, respectively), mitigating concerns over exposure to this protein in food and feed from soybean varieties tolerant to *Roundup*^®^ herbicides.

**Electronic supplementary material:**

The online version of this article (10.1007/s11248-018-0092-z) contains supplementary material, which is available to authorized users.

## Introduction

### Adoption of transgenic soybean

Since their introduction in 1996, genetically engineered (GE, also referred to as GM or GMO) crops have delivered substantial agronomic, environmental, economic, health and social benefits to both farmers and society at large (ISAAA 2016). The 109-fold increase in acreage of GE crops during the 1996–2016 period of their commercialization is indicative of the benefits realized by both large and small farmers worldwide. In 2016, as much as 78% of soybean acres were planted with GE varieties resulting in the highest adoption rate of GE crops globally (ISAAA 2016). These varieties include products with both single and stacked GE traits. In 2016, herbicide-tolerant soybean was planted on 68 million ha, whereas varieties with stacked transgenes (herbicide-tolerance and insect-protected traits) were grown on 23.4 million ha worldwide (ISAAA 2016). As more GE traits become available to farmers, the proportion of varieties with stacked transgenes will become more prevalent.

*Roundup Ready*™ herbicide tolerant GE traits have been widely cultivated for over 20 years. *Roundup Ready*™ soybean exhibit tolerance to glyphosate, the active ingredient of the *Roundup*^®^ family of agricultural herbicides. *Roundup Ready*™ varieties contain a gene derived from a naturally-occurring soil microbe, *Agrobacterium sp*. strain CP4 that encodes the *cp4 epsps* gene that confers tolerance to glyphosate. Five-enolpyruvylshikimate-3-phosphate synthase (EPSPS), an enzyme from the shikimate pathway, catalyzes the reversible reaction of phosphoenolpyruvate (PEP) and 5-hydroxyl of shikimate-3-phosphate (S3P) to form EPSP and phosphate (Padgette et al. 1995).

### Safety of CP4 EPSPS

The food and feed safety of the proteins produced by GE crops are assessed using a two-tiered, weight-of-evidence approach (Delaney et al. 2008; Codex Alimentarius 2009; Hammond et al. 2013). The first tier provides a weight of the evidence to support protein safety by assessing: history of safe use, bioinformatics analysis, mode of action, in vitro digestibility, heat stability, expression level and dietary intake (Delaney et al. 2008). Based on the first-tier assessment, no hazard was identified for CP4 EPSPS (Harrison et al. 1996; Nair et al. 2002). This first-tier data included: (1) bioinformatic analysis showing no significant structural similarities between CP4 EPSPS and proteins associated with allergy or toxicity, and (2) susceptibility of CP4 EPSPS to rapid degradation by both pepsin and pancreatin, supporting a conclusion that this protein is unlikely to be allergic or toxic and that no meaningful exposure to intact CP4 EPSPS will occur through ingestion of food or feed from crops expressing this protein (Harrison et al. 1996).

On a case-by-case basis, confirmatory second tier of testing can be used to further assess the potential for mammalian toxicity using an appropriate animal model (e.g., in vivo toxicology study). Even though no hazard was identified in the first-tier assessment, an acute toxicity study with mice was conducted with CP4 EPSPS for further safety assurance. No adverse effects were observed when mice received an acute dose of 572 mg/kg body weight by oral gavage, a dose much higher than anticipated human exposures from foods potentially containing the CP4 EPSPS.

### Variability of protein expression

Both conventional and GE crops are typically tested across a large number of environments, and those that show superior results and performance stability are brought to market (Privalle et al. 2012; Prado et al. 2014; Glenn et al. 2017). For GE crops, additional comprehensive assessment of potential food, feed and environmental risks are completed prior to commercialization as part of requirements by global regulatory agencies (Cellini et al. 2004; König et al. 2004; EFSA 2006; Paoletti et al. 2008; Codex Alimentarius 2009; Hoekenga et al. 2013). As part of this risk assessment both the potential hazard of the introduced protein, as well as the exposure is evaluated. The assessment of exposure is in part based on protein expression levels and factors that might influence this variation.

It has been reported that the protein expression levels (endogenous and GE) are highly variable depending on genotypic (Trtikova et al. 2015) and environmental factors (Nguyen and Jehle 2007; Jamal et al. 2009). Genotypic factors that influence variation in protein expression can be associated with the genes controlling the protein or the genetic makeup of the plant. A large number of genes are present in multiple copies throughout genome (Stranger et al. 2007; Springer et al. 2009; Zheng et al. 2011) and this variation has been associated with differences in expression levels (Stranger et al. 2007; Springer et al. 2009). Increased copy number of a gene can correlate with protein expression levels either positively (Gendloff et al. 1990; Hobbs et al. 1990, 1993; Falco et al. 1995; James et al. 2002; Halfhill et al. 2003; Stranger et al. 2007) or negatively (Hobbs et al. 1990, 1993; James et al. 2002; Stranger et al. 2007). Furthermore, protein expression often varies in different plant tissues (Padgette et al. 1995; Down et al. 2001; Nguyen and Jehle 2007; Gampala et al. 2017; Matthews et al. 2017). Even the same plant tissue sampled at different days or developmental stages may show differences in expression (Gendloff et al. 1990; Down et al. 2001; Nguyen and Jehle 2007; Matthews et al. 2017).

In addition to genotypic factors, the environment can influence the expression of both endogenous and GE proteins. Environmental conditions (e.g., temperature, light, water availability, nutrients, environmental stresses) associated with different seasonal and geographical variables have been shown to impact plant growth and development influencing protein expression (Down et al. 2001; Heck et al. 2005; Nguyen and Jehle 2007; Jamal et al. 2009; Trtikova et al. 2015; Gampala et al. 2017; Geng et al. 2017). Plant-to-plant variation within the same genetic background has been observed (Down et al. 2001; Nguyen and Jehle 2007) as even subtle environmental differences can influence variation in protein expression (Raser and O’Shea 2005).

The objectives of this study were: (1) to evaluate the variability of CP4 EPSPS expression in different soybean tissues collected across diverse germplasm grown in Argentina, Brazil and the USA, (2) to use variance component analysis to identify the factors that affect CP4 EPSPS expression, (3) to assess if the observed variability substantially impacts estimates of food and feed exposure to CP4 EPSPS and risk assessment endpoints like margins of exposure (MOE) and (4) to demonstrate the redundancy of the protein expression data required for global regulatory approvals of the products containing the *cp4 epsps* transgene.

## Materials and methods

### Field trials

The tissue-specific expression of CP4 EPSPS used in this study was generated across 14 different field trials: two in Argentina, seven in Brazil and five in the USA (S-Table 1). The trials were conducted across nine seasons (from 2007 to 2014/2015) and 22 states/provinces representing a total of 74 environments (10 in Argentina, 33 in Brazil and 31 in the USA) in regions suitable for commercial soybean production. All 14 field trials were planted using a randomized complete block design. The trials conducted in 2007 in the USA and in 2007/2008 in Argentina had three replications, whereas all the other field trials had four replications. These trials were selected for this study because the protein expression data associated with them were submitted to global regulatory agencies in support of import or cultivation approvals of products containing the *cp4 epsps* transgene.

Soybeans grown across the 14 trials were of different maturity groups (ranging from MG 3 to MG 9) depending on the region of adaptation. All the varieties grown in the USA belonged to MG 3–4, whereas those grown in Argentina and Brazil had a wider range of maturity groups (MG 3–6 and MG 5–9, respectively). The entries considered in this study were *Roundup Ready*™ soybeans that exhibit tolerance to the *Roundup*^*®*^ family of agricultural herbicides (Monsanto Company, St. Louis, MO, USA). In addition to the glyphosate-tolerance trait, some entries were stacked with other transgenes: insect-protected traits, nutritionally-enhanced traits and/or other herbicide-tolerance traits (S-Tables 1, 2). The glyphosate-tolerance trait was stacked with one, two or three other GE traits making two-way, three-way, or four-way stacks, respectively. Most of the entries evaluated in this study (91%) contained the *cp4 epsps* gene at one locus, whereas two trials (USA 2009 and Argentina 2013/2014) included a stacked product that contained *cp4 epsps* gene at two loci (originating from MON 89,788 and MON 87,705) (S-Table 2). All CP4 EPSPS entries were treated with *Roundup*^*®*^ herbicide at the prescribed rate during the season.

Plant tissue samples were collected throughout the season from early development at V3 to full maturity at R8 stage (Pedersen 2004). Leaf samples (leaf 1, leaf 2, leaf 3 and leaf 4) represented the youngest fully expanded trifoliates collected at four developmental stages V3–V5, V4–V9, R1–R3 and R3–R6, respectively. The root samples were collected at the R6 stage and were thoroughly washed of soil. The forage samples represented the whole above ground plant (including stems, leaves and pods) at the R6 stage. The leaf, root and forage tissues were collected and stored on dry ice within 30 min of sample collection. Seed was harvested at maturity (R8) and kept at ambient temperature prior to preparation for analysis.

### ELISA for CP4 EPSPS

Soybean tissue samples were ground in a grinder for approximately 1 min. The CP4 EPSPS was extracted from about 100 mg of each ground soybean tissue by using a Harbil Mixer (Fluid Management, Inc, Wheeling, IL, USA) with about 10 ml (1–100 tissue to buffer ratio was determined to be optimal) of trisborate buffer consisting of 0.1 M Tris, 0.1 M Na_2_B_4_O_7_, 0.005 M MgCl_2_, 0.05% (v/v) Tween20 and 0.2% (w/v) L-ascorbic acid (pH 7.8). Insoluble material was removed from soybean tissue extract using a 16 mm × 4″ serum filter (Cat. No.: 02-681-51, Fisher Scientific, Pittsburgh, PA, USA). The tissue extracts were stored at − 80 °C until analysis.

An enzyme-linked immunosorbent assay **(**ELISA) was developed and validated for the detection of CP4 EPSPS. Quantification of CP4 EPSPS was accomplished by interpolation from the logistic curve-fits of the purified CP4 EPSPS standard. The protein standard was produced by fermentation of *E. coli*. The protein standard (97% purity by sodium dodecyl sulfate polyacrylamide gel electrophoresis and densitometric analysis) was stored in a buffer solution containing 50 mM Tris–Cl, pH 7.5, 25% (v/v) glycerol, 50 mM KCl and 2 mM DTT. The identity of the protein was confirmed by N-terminal sequencing using 15 automated cycles of Edman degradation chemistry (Hunkapiller et al. 1983), and peptide mass fingerprint analysis (64% coverage) using MALDI-TOF mass spectrometry. The specific activity of the enzyme was 4.86 Units/mg CP4 EPSPS based on a spectrophotometric assay for released inorganic phosphate (Lanzetta et al. 1979). For the CP4 EPSPS ELISA, the antibody sandwich (capture antibody from mouse) was detected with goat anti–CP4 EPSPS horseradish peroxidase conjugate followed by development with horseradish peroxidase substrate and the enzymatic reaction was terminated by the addition of 6 M H_3_PO_4_. A buffer blank, a negative control and a positive control were also included on every ELISA plate. Positive control was GE soybean tissue extract that contained CP4 EPSPS and the negative control was conventional soybean tissue extract. All ELISA plates were analyzed on a SPECTRAmax Plus 384 (Molecular Devices, Sunnyvale, CA) microplate spectrophotometer, using a dual wavelength (450 and 620 nm as reference) detection method. For all data that was greater than or equal to the limit of quantification, the amount of CP4 EPSPS was estimated by interpolation from the standard curve and the level in the tissues was calculated on a µg/g fresh weight (fw) basis. The moisture content was measured using a moisture analyzer system (Mettler-Toledo, LLC, Columbus, OH, USA) and was used to convert the fresh weight value of to dry weight of the CP4 EPSPS expression. This conversion was done considering tissue moisture levels obtained for each tissue type at each location.

### Margin of exposure (MOE)

A margin of exposure is calculated by dividing the value for no observed adverse effect level (NOAEL) obtained from an appropriate toxicology study by the estimated intake value of the evaluated substance (Eaton and Gilbert 2008).

The MOEs for this protein were calculated by dividing the highest tested dose from an acute toxicity study with CP4 EPSPS in which no adverse effect was noted (Harrison et al. 1996) by the estimated CP4 EPSPS intake value. The acute study is considered an appropriate surrogate for food safety of this protein because toxic proteins tend to act acutely (Sjoblad et al. 1992; Hammond and Fuchs 1998; Pariza and Johnson 2001). The protein intake value used to calculate a MOE was obtained by multiplying CP4 EPSPS expression levels (µg/g fresh weight) in soybean seed by soybean consumption data (USDA 2017). Fresh weight expression values were calculated by multiplying the dry weight protein expression by a correction factor of 0.898 to account for an average 10.2% moisture content in the soybean seed harvested in these trials. The MOE values were calculated based on the overall average and the maximum CP4 EPSPS expression values across all field trials.

### Statistical methods

Statistical analysis was conducted using ELISA results from a total of 3989 soybean tissue samples. The SAS procedure PROC MEANS was used to calculate sample mean, standard deviation and standard error for target variables (SAS 2012). The_ENREF_1 differences among the levels for each of target variable were evaluated at the 5% significance level.

The following linear mixed models were used for estimation of genotypic variables including *cp4 epsps* locus number (one or two), soybean maturity groups (MG 3–4, MG 5–6 or MG 8–9), singles versus stacks, number of GE traits (2-way, 3-way or 4-way stack) and the type of GE traits in stacks (herbicide-tolerance, insect-protection or nutritionally-enhanced):

Locus number:

$${\text{Y}}_{\text{ijklm}} = \mu + {\text{M}}_{\text{i}} + {\text{T}}\left( {\text{M}} \right)_{{{\text{j}}\left( {\text{i}} \right)}} + {\text{E}}_{\text{k}} + {\text{M\,*\,E}}_{\text{ik}} + {\text{E\,*\,T}}\left( {\text{M}} \right)_{{{\text{kj}}\left( {\text{i}} \right)}} + \varepsilon_{\text{ijklm}}$$

where ${\text{Y}}_{\text{ijklm}}$ is the CP4 EPSPS expression of locus number i, trait j, field trial k, location l and observation m; $\mu$ is the overall mean; ${\text{M}}_{\text{i}}$ is the fixed effect of the ith locus number; ${\text{T}}\left( {\text{M}} \right)_{{{\text{j}}\left( {\text{i}} \right)}}$ is the fixed effect of the jth trait nested within the ith locus number; ${\text{E}}_{\text{k}}$ is the random effect of the kth location; ${\text{M\,*\,E}}_{\text{ik}}$ is the random effect of the interaction between the ith locus number and the lth location; ${\text{E\,*\,T}}\left( {\text{M}} \right)_{{{\text{kj}}\left( {\text{i}} \right)}}$ is the random effect of the interaction between the kth field trial and jth trait nested within the ith locus number; and $\varepsilon_{\text{ijklm}}$ is the residual error.

Maturity group:

$${\text{Y}}_{\text{ijklm}} = \mu + {\text{M}}_{\text{i}} + {\text{T}}_{\text{j}} + {\text{M}}\, *\,{\text{T}}_{\text{ij}} + {\text{S}}_{\text{k}} + {\text{E}}\left( {\text{S}} \right)_{{{\text{l}}\left( {\text{k}} \right)}} + {\text{M}}\, *\,{\text{E}}\left( {\text{S}} \right)_{{{\text{il}}\left( {\text{k}} \right)}} + {\text{T}}\, *\,{\text{E}}\left( {\text{S}} \right)_{{{\text{jl}}\left( {\text{k}} \right)}} + \varepsilon_{\text{ijklm}}$$

where ${\text{Y}}_{\text{ijklm}}$ is the CP4 EPSPS expression of maturity group i, trait j, field trial k, location l and observation m; $\mu$ is the overall mean; ${\text{M}}_{\text{i}}$ is the fixed effect of the ith maturity group; ${\text{T}}_{\text{j}}$ is the fixed effect of the jth trait; ${\text{M\,*\,T}}_{\text{ij}}$ is the random effect of the interaction between the ith maturity group and the jth trait; ${\text{S}}_{\text{k}}$ is the random effect of the kth field trial; ${\text{E}}\left( {\text{S}} \right)_{{{\text{l}}\left( {\text{k}} \right)}}$ is the random effect of the lth location nested within the kth field trial; ${\text{M\,*\,E}}\left( {\text{S}} \right)_{{{\text{il}}\left( {\text{k}} \right)}}$ is the random effect of the interaction between the ith maturity group and the lth location nested within the kth field trial; ${\text{T\,*\,E}}\left( {\text{S}} \right)_{{{\text{jl}}\left( {\text{k}} \right)}}$ is the random effect of the interaction between the jth trait and the lth location nested within the kth field trial; and $\varepsilon_{\text{ijklm}}$ is the residual error.

Singles versus stacks:

$${\text{Y}}_{\text{ijklm}} = \mu + {\text{M}}_{\text{i}} + {\text{T}}\left( {\text{M}} \right)_{{{\text{j}}\left( {\text{i}} \right)}} + {\text{S}}_{\text{k}} + {\text{E}}\left( {\text{S}} \right)_{{{\text{l}}\left( {\text{k}} \right)}} + {\text{M\,*\,E}}\left( {\text{S}} \right)_{{{\text{il}}\left( {\text{k}} \right)}} + {\text{T\,*\,E}}\left( {\text{S}} \right)_{{{\text{jl}}\left( {\text{k}} \right)}} + \varepsilon_{\text{ijklm}}$$

where ${\text{Y}}_{\text{ijklm}}$ is the CP4 EPSPS expression of trait number (i.e., single or stack) i, trait j, field trial k, location l and observation m; $\mu$ is the overall mean; ${\text{M}}_{\text{i}}$ is the fixed effect of the ith trait number; ${\text{T}}\left( {\text{M}} \right)_{{{\text{j}}\left( {\text{i}} \right)}}$ is the random effect of the jth trait nested within the ith trait number; ${\text{S}}_{\text{k}}$ is the random effect of the kth field trial; ${\text{E}}\left( {\text{S}} \right)_{{{\text{l}}\left( {\text{k}} \right)}}$ is the random effect of the lth location nested within the kth field trial; ${\text{M\,*\,E}}\left( {\text{S}} \right)_{{{\text{il}}\left( {\text{k}} \right)}}$ is the random effect of the interaction between the ith trait number and the lth location nested within the kth field trial; ${\text{T\,*\,E}}\left( {\text{S}} \right)_{{{\text{jl}}\left( {\text{k}} \right)}}$ is the random effect of the interaction between the jth trait and the lth location nested within the kth field trial; and $\varepsilon_{\text{ijklm}}$ is the residual error.

Number of GE traits in stacks:

$${\text{Y}}_{\text{ijklm}} = \mu + {\text{M}}_{\text{i}} + {\text{T}}\left( {\text{M}} \right)_{{{\text{j}}\left( {\text{i}} \right)}} + {\text{S}}_{\text{k}} + {\text{E}}\left( {\text{S}} \right)_{{{\text{l}}\left( {\text{k}} \right)}} + {\text{M\,*\,E}}\left( {\text{S}} \right)_{{{\text{il}}\left( {\text{k}} \right)}} + {\text{T\,*\,E}}\left( {\text{S}} \right)_{{{\text{jl}}\left( {\text{k}} \right)}} + \varepsilon_{\text{ijklm}}$$

where ${\text{Y}}_{\text{ijklm}}$ is the CP4 EPSPS expression of trait number i, trait j, field trial k, location l and observation m; $\mu$ is the overall mean; ${\text{M}}_{\text{i}}$ is the fixed effect of the ith trait number; ${\text{T}}\left( {\text{M}} \right)_{{{\text{j}}\left( {\text{i}} \right)}}$ is the random effect of the jth trait nested within the ith trait number; ${\text{S}}_{\text{k}}$ is the random effect of the kth field trial; ${\text{E}}\left( {\text{S}} \right)_{{{\text{l}}\left( {\text{k}} \right)}}$ is the random effect of the lth location nested within the kth field trial; ${\text{M\,*\,E}}\left( {\text{S}} \right)_{{{\text{il}}\left( {\text{k}} \right)}}$ is the random effect of the interaction between the ith trait number and the lth location nested within the kth field trial; ${\text{T\,*\,E}}\left( {\text{S}} \right)_{{{\text{jl}}\left( {\text{k}} \right)}}$ is the random effect of the interaction between the jth trait and the lth location nested within the kth field trial; and $\varepsilon_{\text{ijklm}}$ is the residual error.

GE trait type:

$${\text{Y}}_{{{\text{ijklm}}}} = \mu + {\text{M}}_{{\text{i}}} + {\text{T}}\left( {\text{M}} \right)_{{{\text{j}}\left( {\text{i}} \right)}} + {\text{S}}_{{\text{k}}} + {\text{E}}\left( {\text{S}} \right)_{{{\text{l}}\left( {\text{k}} \right)}} + {\text{M}}*{\text{E}}\left( {\text{S}} \right)_{{{\text{il}}\left( {\text{k}} \right)}} + {\text{T}}*{\text{E}}\left( {\text{S}} \right)_{{{\text{jl}}\left( {\text{k}} \right)}} + \varepsilon _{{{\text{ijklm}}}}$$

where ${\text{Y}}_{\text{ijklm}}$ is the CP4 EPSPS expression of trait type i, trait j, field trial k, location l and observation m; $\mu$ is the overall mean; ${\text{M}}_{\text{i}}$ is the fixed effect of the ith trait type; ${\text{T}}\left( {\text{M}} \right)_{{{\text{j}}\left( {\text{i}} \right)}}$ is the random effect of the jth trait nested within the ith trait type; ${\text{S}}_{\text{k}}$ is the random effect of the kth field trial; ${\text{E}}\left( {\text{S}} \right)_{{{\text{l}}\left( {\text{k}} \right)}}$ is the random effect of the lth location nested within the kth field trial; ${\text{M\,*\,E}}\left( {\text{S}} \right)_{{{\text{il}}\left( {\text{k}} \right)}}$ is the random effect of the interaction between the ith trait type and the lth location nested within the kth field trial; ${\text{T\,*\,E}}\left( {\text{S}} \right)_{{{\text{jl}}\left( {\text{k}} \right)}}$ is the random effect of the interaction between the jth trait and the lth location nested within the kth field trial; and $\varepsilon_{\text{ijklm}}$ is the residual error.

The following linear mixed models were used for estimation of environmental variables (country and season):

Country:

$${\text{Y}}_{\text{ijkl}} = \mu + {\text{M}}_{\text{i}} + {\text{T}}_{\text{j}} + {\text{M\,*\,T}}_{\text{ij}} + {\text{E}}\left( {\text{M}} \right)_{{{\text{k}}\left( {\text{i}} \right)}} + {\text{T\,*\,E}}\left( {\text{M}} \right)_{{{\text{jk}}\left( {\text{i}} \right)}} + \varepsilon_{\text{ijkl}}$$

where ${\text{Y}}_{\text{ijkl}}$ is the CP4 EPSPS expression of country i, trait j, location k and observation l; $\mu$ is the overall mean; ${\text{M}}_{\text{i}}$ is the fixed effect of the ith country; ${\text{T}}_{\text{j}}$ is the fixed effect of the jth trait; ${\text{M\,*\,T}}_{\text{ij}}$ is the random effect of the interaction between the ith country and the jth trait; ${\text{E}}\left( {\text{M}} \right)_{{{\text{k}}\left( {\text{i}} \right)}}$ is the random effect of the kth location nested within the ith country; ${\text{T\,*\,E}}\left( {\text{M}} \right)_{{{\text{jk}}\left( {\text{i}} \right)}}$ is the random effect of the interaction between the jth trait and the kth location nested within the ith country; and $\varepsilon_{\text{ijkl}}$ is the residual error.

Season:

$${\text{Y}}_{\text{ijkl}} = \mu + {\text{M}}_{\text{i}} + {\text{T}}_{\text{j}} + {\text{M\,*\,T}}_{\text{ij}} + {\text{E}}\left( {\text{M}} \right)_{{{\text{k}}\left( {\text{i}} \right)}} + {\text{T\,*\,E}}\left( {\text{M}} \right)_{{{\text{jk}}\left( {\text{i}} \right)}} + \varepsilon_{\text{ijkl}}$$

where ${\text{Y}}_{\text{ijkl}}$ is the CP4 EPSPS expression of season i, trait j, location k and observation l; $\mu$ is the overall mean; ${\text{M}}_{\text{i}}$ is the fixed effect of the ith season; ${\text{T}}_{\text{j}}$ is the fixed effect of the jth trait; ${\text{M\,*\,T}}_{\text{ij}}$ is the random effect of the interaction between the ith season and the jth trait; ${\text{E}}\left( {\text{M}} \right)_{{{\text{k}}\left( {\text{i}} \right)}}$ is the random effect of the kth location nested within the ith season; ${\text{T\,*\,E}}\left( {\text{M}} \right)_{{{\text{jk}}\left( {\text{i}} \right)}}$ is the random effect of the interaction between the jth trait and the kth location nested within the ith season; and $\varepsilon_{\text{ijkl}}$ is the residual error.

### Variance component analysis

Variance component analysis (VCA) was used to assess the amount of variation in CP4 EPSPS expression that is associated with key genotypic and environmental variables. The following random effects model was used:

$$Y = \mu + {\text{G}} + {\text{E}} + {\text{G}}\,*\,{\text{E}} + \varepsilon ,$$

where Y is the CP4 EPSPS expression, μ is the overall mean, G is set of genotypic variables, E is a set of environmental variables, G * E is the interaction between genotypic and environmental variables and ε is residual error. SAS PROC MIXED was used to estimate the covariance parameters for all random effects appearing in the model. The variance component parameters for all effects (genotypic, environmental and genotypic by environmental) were divided by the total variance to get the variance proportion for each.

## Results and discussion

The efficacy of the *cp4 epsps* gene that confers tolerance to glyphosate across commercial *Roundup Ready*™ varieties has been uniform and consistent (Nair et al. 2002). The efficacy of the *cp4 epsps* gene for varieties used in this study was verified by treating the plots with *Roundup*^*®*^ herbicide. No plant injury or mortality was observed in any of the treated plots (data not shown) which indicated the efficacy of *cp4 epsps* gene and that the protein is being expressed correctly.

The assessment of CP4 EPSPS expression was based on robust data collected across diverse genetic and environmental factors. The level of CP4 EPSPS expression was evaluated in a total of 3989 *Roundup Ready*™ soybean samples. Four plant tissues were collected (i.e., leaf at different development stages, root, forage and seed) from diverse glyphosate-tolerant GE varieties. All varieties contained the *cp4 epsps* gene either as a single product or stacked with one, two or three other GE traits (S-Table 1). The stacked products included entries where the *cp4 epsps* gene was combined with other herbicide-tolerance (MON 87708), insect-protected (MON 87701, MON 87751) and/or nutritionally-enhanced (MON 87705, MON 87769) traits (S-Tables 1, 2). Most of the entries had *cp4 epsps* gene at one locus, but some had it at two loci (when both MON89788 and MON 87705 were included). Samples were collected from 14 different field trials in Argentina, Brazil and the USA representing diverse locations across 22 states/provinces. The trials were conducted over nine seasons (2007–2014/2015), totaling 74 different environmental conditions. Soybean varieties were adapted to these different regions and ranged from MG 3 to MG 9. This diversity of genetic and environmental factors provided an opportunity for a very comprehensive evaluation of CP4 EPSPS expression and factors that may influence it.

### Genotypic factors

Several genotypic sources of variation in CP4 EPSPS expression were considered in this research. Some were associated with the GE traits like number of *cp4 epsps* loci, number and type of stacked GE traits. Others were associated with genetic factors like tissue type (which will have differential patterns of expression unique to each tissue) or diverse soybean varieties included in these field trials.

**Fig. 1:**
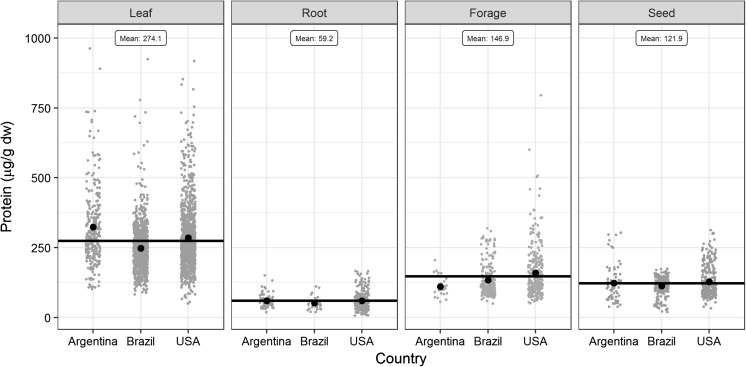
Variation of single locus CP4 EPSPS expression (µg/g dw) observed for different growing regions. Each dot represents a data point of CP4 EPSPS expression from either Argentina, Brazil or the United States, whereas the horizontal line indicates mean value per tissue type. The leaf data represents all growth stages of leaf analyzed

The number of copies of a gene can influence expression levels (Hobbs et al. 1990, 1993; James et al. 2002; Halfhill et al. 2003; Stranger et al. 2007; Springer et al. 2009) and may result in differences associated with phenotypic characteristics like disease resistance in soybean (Cook et al. 2012) or heterosis in maize (Springer et al. 2009). In this study, all field trials included entries that contained a single *cp4 epsps* locus, whereas two field trials also included entries with two *cp4 epsps* loci. Entries containing two *cp4 epsps* loci had higher protein expression that tended to show approximately twice the expression observed for those containing a single *cp4 epsps* locus across all tissue types (S-Fig. 1). This is in agreement with those studies that show positive correlation between gene copy number and expression levels (Gendloff et al. 1990; Falco et al. 1995; Halfhill et al. 2003). The same researchers also point out that this correlation can be negative for some genes or events. Thus, our results regarding the additive gene action associated with *cp4 epsps* is applicable for evaluated events, but cannot be extrapolated to other traits.

Even though the differences in expression between entries with one and two *cp4 epsps* loci were significant, tolerance to *Roundup*^®^ herbicide applications that followed label requirements was observed across all trials and for all CP4 EPSPS entries regardless of locus number.

To provide tolerance to *Roundup*^®^ herbicide, the CP4 EPSPS is expressed throughout the plant and across developmental stages. Previous studies showed variation in protein expression associated with different plant tissues (Padgette et al. 1995; Down et al. 2001; Nguyen and Jehle 2007; Gampala et al. 2017; Matthews et al. 2017) and different developmental stages (Gendloff et al. 1990; Down et al. 2001; Nguyen and Jehle 2007). In our research, for a single locus entries, no significant difference in CP4 EPSPS expression was observed among leaf tissues collected at four different stages of plant development (i.e., V3–V5, V4–V9, R1–R3 and R3–R6) with the mean values ranging from 254.4 to 290.7 μg/g (S-Fig. 1). The lowest CP4 EPSPS expression for single locus entries was detected for root tissue (54.4 μg/g), whereas the values for forage and seed tissues were similar (157.4 and 123.5 μg/g, respectively), but were both significantly lower compared to those observed for leaf tissue. These results are similar to previously reported studies on CP4 EPSPS expression (Padgette et al. 1995; Nair et al. 2002; Heck et al. 2005; Gampala et al. 2017).

Similar trends for the relative expression levels in different tissues were observed for entries with two *cp4 epsps* loci. Numerically, leaf tissues had the highest CP4 EPSPS expression (ranging from 436.5 to 650.6 μg/g), followed by forage (340.2 μg/g), seed (207.1 μg/g) and root tissue averaging 126.5 μg/g (S-Fig. 1). Most of the leaf comparisons were not significantly different, with the exception of leaf 1 which had significantly lower expression values than leaf 4.

Factors associated with genetic background can be evaluated as potential sources of variation for protein expression (Trtikova et al. 2015; Geng et al. 2017). Varieties considered in this study were grouped into three maturity clusters ranging from MG 3 (adapted to temperate climate) to MG 9 (adapted for tropics). This wide range represents genetic diversity of tested entries, as the varieties of different maturity were developed in different breeding programs within the regions of adaptation. No significant differences in CP4 EPSPS expression were observed among the three maturity clusters for any of the tissue types except for leaf 3 where MG 8–9 had significantly lower CP4 EPSPS expression (Table 1). Similar results were obtained when analysis was conducted for soybean varieties included in this study (data not shown). This indicates that genetic background of different varieties or the time required to reach plant maturity were not important contributors to variability of CP4 EPSPS expression. This observation that genetic background and maturity of soybean varieties do not significantly impact variability in expression of CP4 EPSPS is comparable with results from studies where expression of several non-transgenic soybean proteins was evaluated (Geng et al. 2017).

**Table 1:** Comparisons for genotypic factors and their effect on single locus CP4 EPSPS expression (µg/g dw)

Genotypic factors	Leaf 1	Leaf 2	Leaf 3	Leaf 4	Root	Forage	Seed
*Maturity groups*							
MG 3–MG 4	238.2	287.8	306.5a	275.7	53.3	168.8	109.8
MG 5–MG 6	289.4	288.7	258.8a	309.0	50.5	139.5	138.4
MG 8–MG 9	247.2	274.4	204.8b	263.5	62.6	147.5	137.2
Differences^a^	NS	NS	*	NS	NS	NS	NS
*Single versus stacks with other GE traits*							
*cp4 epsps* single	263.2	304.1	287.8	342.5	79.0	169.4	152.9
*cp4 epsps* stacks	259.6	279.7	264.7	258.0	42.8	149.8	108.6
Differences^a^	NS	NS	NS	NS	NS	NS	NS
*Number of GE traits in stacks*							
2-Way stack	243.5	294.3	249.1	249.5	43.5	126.1	107.4
3-Way stack	276.6	261.8	350.9	261.1	64.5	214.5	130.8
4-Way stack	284.3	331.1	350.9	283.9	21.7	181.7	116.9
Differences^a^	NS	NS	NS	NS	NS	NS	NS
*Type of GE traits in stacks*							
Herbicide-tolerance	230.0	268.6	219.9	225.9	34.3	107.2	98.7
Insect-protection	309.2	302.2	297.2	267.4	45.8	162.4	118.1
Nutritionally-enhanced	212.4	246.0	227.9	243.7	46.5	148.0	115.2
Differences^a^	NS	NS	NS	NS	NS	NS	NS

*NS* not significant differences

^a^Means within a column for each genotypic factor followed by different letters are significantly different at 0.05 significance level: *significant differences

With development of new GE traits, the adoption of stacked products in the marketplace steadily increases across crops. In Brazil, for example, soybean varieties with herbicide-tolerance stacked with insect-protected trait increased from 2.2 million ha in 2013/2014 season to 20.2 million ha in 2015/2016 (ISAAA 2016). In our study, some of the entries contained only the *cp4 epsps* gene (single GE trait), whereas others had the *cp4 epsps* gene stacked with one, two or three different GE traits (S-Tables 1, 2). Comparisons between single and stacked GE products indicated that there were no significant differences in CP4 EPSPS expression levels for any of the evaluated tissue types (Table 1). Furthermore, no significant differences were observed among stacked products regardless of number of GE traits for any of the evaluated tissues. Generally, our data indicated that the presence of the other transgene(s) did not influence the CP4 EPSPS expression levels. Similarly, others showed comparable results between single and stacked products for soybean, maize and cotton products they evaluated (Gampala et al. 2017).

Entries stacked with *cp4 epsps* gene evaluated in this study included three types of GE traits (tolerance to dicamba herbicide, insect-protection and traits that provide nutritional enhancement). There were no differences in CP4 EPSPS expression observed among these three groups of stacked products for any of the tissue types (Table 1) indicating that *cp4 epsps* gene did not interact with a transgene that provides similar function (i.e., dicamba herbicide-tolerance), nor with those that provide different functions (i.e., insect-protection and nutritionally-enhanced traits).

### Environmental factors

When protein expression is evaluated in different seasons and/or locations (Nair et al. 2002; Nguyen and Jehle 2007; Geng et al. 2017), it provides an opportunity to assess the potential influence of environmental factors on expression levels. In our study, the field testing was conducted in 4–8 locations per trial, from the 2007 to the 2014/2015 growing season, representing a diverse range of environmental conditions across North and South American regions where soybean is typically grown. The trials were conducted in three countries: Argentina, Brazil and the USA, with two trials grown in Argentina, seven in Brazil and five in the USA. These diverse regions are associated with climate conditions ranging from continental to tropical. Despite this geographic diversity, significant differences were detected for expression levels only in leaf 3 tissue across countries, with no differences for the other leaf tissues, root, forage and seed tissues (Table 2). Despite this lack of significant differences, the high variability of CP4EPSPS expression within each region is noteworthy for the evaluated tissues (Fig. 1).

**Table 2:** Comparisons for environmental factors and their effect on single locus CP4 EPSPS expression (µg/g dw)

Environment	Leaf 1	Leaf 2	Leaf 3	Leaf 4	Root	Forage	Seed
*Countries*							
Argentina	410.1	301.6	255.7ab	337.1	38.0	124.7	111.4
Brazil	225.9	268.5	220.6b	266.3	63.3	125.0	127.9
USA	241.5	293.8	330.8a	286.9	54.9	182.7	128.1
Differences^a^	NS	NS	*	NS	NS	NS	NS
*Seasons* ^*b*^							
2007	248.7 b	265.9	304.1b	239.9	40.7	119.8c	103.8
2007/2008	369.3 a	322.2	203.1c	344.4	41.0	119.1c	116.1
2008/2009	207.0 b	196.7	233.8bc	337.1	–	263.0ab	144.9
2009	186.8 b	332.5	258.1bc	334.1	66.6	159.8c	129.8
2009/2010	196.2 b	182.6	203.8bc	168.7	–	–	–
2012/2013	304.1 ab	362.4	282.8bc	291.6	66.1	156.3bc	145.5
2013	299.1 ab	267.9	455.1a	277.9	62.7	273.2a	167.7
2013/2014	245.9 b	264.5	237.2bc	258.1	40.6	180.8bc	90.1
2014/2015	273.5 ab	300.5	253.5bc	291.5	–	104.2c	151.9
Differences^a^	*	NS	*	NS	NS	*	NS

*NS* not significant differences

^a^Means within a column for each environmental factor followed by different letters are significantly different at 0.05 significance level: *significant differences;

^b^See S-Table 1 for information on the specific geography associated with the data for specific seasons

**Fig. 2:**
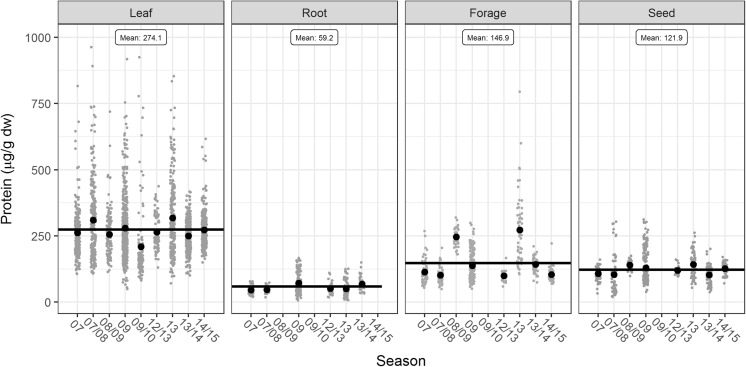
Variation of single locus CP4 EPSPS expression (µg/g dw) observed for different growing seasons. Each dot represents a data point of CP4 EPSPS expression from either 2007, 2007/2008, 2008/2009, 2009, 2009/2010, 2012/2013, 2013 2013/2014 or 2014/2015 season, whereas the horizontal line indicates mean value per tissue type. The leaf data represents all growth stages of leaf analyzed

Similar analysis was performed across nine growing seasons. Most included a single field trial with the exception of 2007, 2009 and 2012/2013 seasons which were represented by two trials each (S-Table 1). Generally, high variability in CP4 EPSPS expression was observed for some of the growing seasons (Fig. 2), with statistically significant differences observed for leaf 1, leaf 3 and forage tissue types (Table 2).

### Variance component analysis

Variance component analysis was used to assess the amount of variation in CP4 EPSPS expression that is associated with key genotypic and environmental variables. The analysis indicated that two genotypic components that contributed the most were *cp4 epsps* locus number and tissue type, whereas genetic background and maturity of soybean varieties, as well as the stacking *cp4 epsps* gene with other biotechnology traits contributed to a lesser extent.

**Fig. 3:**
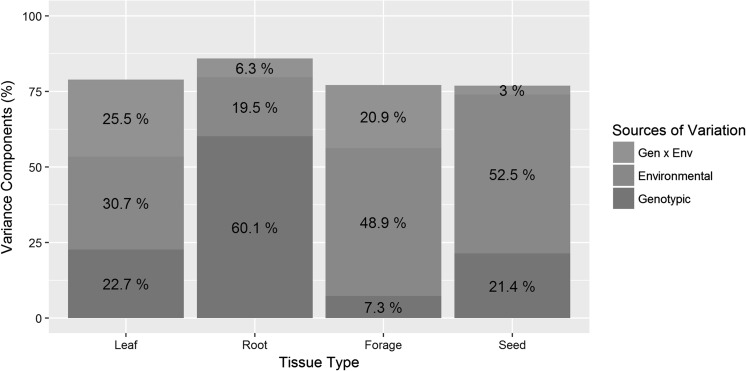
Variance component analysis of single locus CP4 EPSPS expression by tissue types considering environmental, genotypic and genotypic × environmental sources of variation

When two major sources of genotypic variation were removed from the analysis (e.g., the variance components for single locus entries by tissue types), the results showed that environmental variation contributed to 19.5–52.5% of the variation depending on the tissue (Fig. 3). Most of the environmental variation was observed for forage and seed tissues, whereas the least was detected for root tissue. Similarly, others have analyzed transgene expression by tissue types and found that environmental factors contributed the most to protein variation (Gampala et al. 2017). The variation observed in our study is most likely attributable to different abiotic/biotic conditions at each testing environment, as well as micro-environmental conditions within each plot (Raser and O’Shea 2005).

Therefore, some level of environmental variation is expected in any field study as there is variability in meteorological and agronomical factors that cannot be completely eliminated. However, these field trials are designed to control for many of the environmental factors that might influence the crop development (e.g., water, nutrition, protection against weeds, insects and diseases) and thus the variation of protein expression. Results generated in these well-designed trials have been used for assessing food, feed and environmental risks, not only in regions where the trials were conducted, but were applicable to other countries/regions. Consequently, duplicated testing in different world areas, sometimes required by regulatory agencies, is not scientifically justified.

### Safety of CP4 EPSPS

For proteins expressed in GE crops, safety assessments include an evaluation of hazard potential (*e.g*., toxicity) and may also include a risk assessment, that places the hazard assessment data in the context of anticipated exposures to the protein (Codex Alimentarius 2009). No hazards have been identified for CP4 EPSPS (Hammond et al. 1996; Harrison et al. 1996; Hammond and Cockburn 2008), therefore, a further assessment of risk is not needed from a scientific standpoint because there is no risk in the absence of hazard (risk = hazard × exposure). However, estimating the MOE provides further context to enable leveraging the hazard characterization data in an overall risk assessment of CP4 EPSPS. The MOE is calculated as a ratio between the highest tested dose from an appropriate toxicity study with the protein at which no adverse effect was noted and a conservative estimate of human dietary exposure to the protein (protein intake) (Eaton and Gilbert 2008). As CP4 EPSPS expression varies due to environmental or genotypic factors, the MOE estimate will reflect these varying expression values. Conservative estimates are used for exposure assessment (e.g., 100% market penetration/full replacement with evaluated trait, no loss of protein during processing) so that a gross overestimate of human exposure is ensured. Full replacement represents an unrealistic exposure scenario and protein exposure assessments reflect significant overestimates because dietary protein exposure tends to be significantly reduced by the harsh conditions of food processing or cooking (Hammond and Jez 2011). Therefore, slight differences in CP4 EPSPS expression (due to genetic or environmental factors) do not fundamentally change the safety conclusion based on the observed lack of hazard in the toxicological assessment and the robust safety margins that demonstrate no meaningful risk to humans from dietary exposure to proteins produced in GE plants.

The appropriate MOE threshold for drawing safety conclusions from dietary exposure to proteins has not been established, but based on the paradigm for toxic chemicals, the traditional approach is to use a minimum of a 100-fold MOE to estimate the level of exposure to an agent that would be assumed to not adversely impact human health (Faustman and Omenn 2008). This 100-fold MOE accounts for inter-species and inter-individual variability. An even more conservative approach is to include an additional 10-fold safety factor to account for other uncertainties such as sensitive subpopulations or a limited toxicology database resulting in a very conservative MOE of 1000. There are three variables (dose level, protein expression and consumption) that can impact the MOE and by either increasing or decreasing one or more of these variables, the MOE will get larger or smaller. Since the focus of this study was variation of CP4 EPSPS expression, the acute non-toxic dose level of 572 mg/kg body weight (Harrison et al. 1996) and soybean consumption of 2.0 g/kg body weight/day (USDA 2017) were kept constant and only the protein expression variable was adjusted to illustrate the modest differences in MOE values associated with changes in protein expression levels in glyphosate-tolerant soybean across the broad dataset used in this study.

The evaluation of CP4 EPSPS expression in soybean seed across different environments illustrates the inherent variability associated with protein expression in GE crops. The maximum value of CP4 EPSPS expression in soybean seed observed in this study was 343.5 µg/g dry weight with an overall mean value of 124 µg/g dry weight across all field trials representing 74 environments, different number of *cp4 epsps* loci and different trait combinations. The estimated MOE values were approximately 4000 and 11,000 for the maximum and average CP4 EPSPS expression values, respectively. These MOE values are about 4 and 11 times higher than the highly conservative approach of providing adequate human health protection with a MOE of 1,000 for toxic chemicals, whereas CP4 EPSPS is a readily digestible protein unlikely to survive food processing conditions. It can therefore be concluded that the observed variations in CP4 EPSPS expression do not impact the overall safety of GE crop products expressing this protein, because empirical data provide MOE values that are sufficiently large to adequately protect human health. These results apply across GE crop products and are consistent with the conclusion that the overall weight of evidence indicates clearly that evaluated GE products can be used safely in food and feed production (Hammond and Cockburn 2008).

In summary, extensive testing of CP4 EPSPS expression levels was conducted across different soybean tissues collected from field trials grown in 74 diverse environments. Two genotypic factors that contributed to significant differences in expression levels in the plant were number of *cp4 epsps* loci and the type of plant tissue tested. Genotypic factors like genetic background or maturity of soybean varieties tested, stacking the *cp4 epsps* gene with one or more transgenes, or the function of the stacked trait(s) did not significantly impact the CP4 EPSPS expression. Generally, environmental conditions like regions and seasons did not have consistent impact on the level of CP4 EPSPS expression for the evaluated tissue types as non-directional variability was observed. These results are informative for evaluations of products containing *cp4 epsps* transgene as they indicate that: (1) Within each experimental factor (locus number, tissue type, genetic background, number of GE traits, type of GE traits, region and season), CP4 EPSPS expression can be highly variable. (2) Across environmental factors (regions and seasons), variability of CP4 EPSPS expression was not significant or directional. The country or region where the crop is grown is not expected to be important for risk assessment associated with GE protein expression. (3) Stacking *cp4 epsps* with one or more GE traits (e.g., different herbicide-tolerance, insect-protected and/or nutritionally-enhanced traits) did not impact CP4 EPSPS expression. (4) The CP4 EPSPS expression was affected by *cp4 epsps* locus number in a predictable manner. (5) CP4 EPSPS expression levels in the tested tissues showed differences that were consistent across samples from a wide range of environmental field conditions. (6) Product efficacy has been demonstrated with a range of expression values. (7) Variability of CP4 EPSPS expression does not materially affect the MOE calculations that consistently estimate negligible risk associated with consumption of CP4 EPSPS. Product safety has been demonstrated despite variability in CP4 EPSPS expression, as demonstrated by vary large MOEs, thereby making a single trait MOE determination applicable to risk assessments for combined trait products. (8) The protein expression data required for global regulatory approvals of any product containing the *cp4 epsps* transgene, including stacks, is extensive and safety and characterization conclusions on protein expression can be derived using more focused data sets.

The research summarized in this report contributes a large data set that comprehensively describes the effect of several genetic and environmental factors on the variation in expression of an introduced protein, CP4 EPSPS, found in many GE crops (single and combined trait). Importantly, both the safety and the efficacy of this introduced protein that provides glyphosate tolerance, are each not affected by the variability in protein expression, ensuring that consumers can trust the safety of their food and farmers can rely and benefit from the trait efficacy.

## Electronic supplementary material

Below is the link to the electronic supplementary material.

Supplementary material 1 (DOCX 66 kb)
