# Supplementary material for: Variability of CP4 EPSPS expression in genetically engineered soybean (Glycine max L. Merrill)
Source: Transgenic Res. 2018 Sep 1;27(6):511–24. doi: 10.1007/s11248-018-0092-z (PMC6267263; doi:10.1007/s11248-018-0092-z)
Supplement: Supplementary file 1 — Supplementary material 1 (DOCX 66 kb) [file 11248_2018_92_MOESM1_ESM.docx]

**Supplementary Material**

**Variability of CP4 EPSPS Expression in Genetically Engineered Soybean (*Glycine ma*x L. Merrill)**

Parimala Chinnadurai†, Duška Stojšin†, Kang Liu, Gregory E. Frierdich, Kevin C. Glenn, Tao Geng, Adam Schapaugh, Keguo Huang, Andrew E. Deffenbaugh*, Zi L. Liu and Luis A. Burzio

Monsanto Company, 700 Chesterfield Pkwy. West, St. Louis, MO 63017, USA

†These authors contributed equally to this work.

*Author for correspondence:

*Andrew Deffenbaugh*

Tel: +1-636-737-9432

Email: [aedeff@Monsanto.com](mailto:pchinna@Monsanto.com)

**S-Table 1** Details associated with 14 soybean field trials conducted in Argentina, Brazil and the USA across nine seasons

| **Field Trials** | **States / Provinces** | **Products** | **Trait types**^a^ |
| --- | --- | --- | --- |
| **Argentina**  **2007/2008** | Buenos Aires (3)^b^, Cordoba, Santa Fe | MON 89788  MON 89788 x MON 87701 | HT  HT, IP |
| **Argentina**  **2013/2014** | Buenos Aires (4), Santa Fe | MON 89788  MON 87705  MON 89788 x MON 87705 x MON 87708 | HT  HT, NE |
| **Brazil**  **2007/2008** | Mato Grosso, Minas Gerais, Parana, Rio Grande do Sul | MON 89788  MON 89788 x MON 87701 | HT  HT, IP |
| **Brazil**  **2008/2009** | Mato Grosso, Minas Gerais, Parana, Rio Grande do Sul | MON 89788  MON 89788 x MON 87701 | HT  HT, IP |
| **Brazil**  **2009/2010** | Mato Grosso, Minas Gerais, Parana, Rio Grande do Sul | MON 89788  MON 89788 x MON 87701 | HT  HT, IP |
| **Brazil**  **2012/2013** | Mato Grosso, Minas Gerais, Parana, Rio Grande do Sul, Sao Paulo | MON 89788 x MON 87708 | HT |
| **Brazil**  **2012/2013** | Minas Gerais, Parana, Rio Grande do Sul, Sao Paulo | MON 89788  MON 89788 x MON 87708 | HT |
| **Brazil**  **2013/2014** | Bahia, Mato Grosso, Minas Gerais, Parana, Rio Grande do Sul, Sao Paulo | MON 89788  MON 89788 x MON 87708 | HT |
| **Brazil**  **2014/2015** | Bahia, Mato Grosso, Minas Gerais, Parana, Rio Grande do Sul, Sao Paulo | MON 89788  MON 89788 x MON 87701 x MON 87751  MON 89788 x MON 87701 x MON 87708 x MON 87751 | HT  HT, IP |
| **USA**  **2007** | Alabama, Arkansas, Georgia, Illinois, North Carolina | MON 89788  MON 89788 x MON 87701 | HT  HT, IP |
| **USA**  **2007** | Iowa, Michigan, Nebraska, Pennsylvania, Wisconsin | MON 89788  MON 89788 x MON 87769 | HT  HT, NE |
| **USA**  **2009** | Illinois (3), Indiana, Iowa, Kansas,  Missouri, Nebraska | MON 89788  MON 87705  MON 89788 x MON 87705 | HT  HT, NE |
| **USA**  **2009** | Arkansas, Illinois (2), Indiana (2), Iowa, Kansas, Nebraska | MON 89788  MON 89788 x MON 87708 | HT |
| **USA**  **2013** | Arkansas, Illinois, Kansas, North Carolina, Pennsylvania | MON 89788  MON 89788 x MON 87701 x MON 87751  MON 89788 x MON 87701 x MON 87708 x MON 87751 | HT  HT, IP |

^a^ Evaluated GE traits were herbicide-tolerance (HT), insect-protected (IP) and/or nutritionally-enhanced (NE). The herbicide-tolerance CP4 EPSPS trait was either single or stacked with other traits (herbicide-tolerance, insect-protected or nutritionally-enhanced traits).

^b^ If more than one location was planted per state/province, their number is indicated in the parenthesis.

**S-Table 2** Description of the herbicide-tolerance, insect-protected and nutritionally-enhanced GE traits considered in this study

| **Trait** | **Description** |
| --- | --- |
| **MON 89788** | A herbicide-tolerant soybean that produces *5enolpyruvylshikimate-3-phosphate synthase* protein from *Agrobacterium* sp. strain CP4 (CP4 EPSPS) to confer tolerance to glyphosate, the active ingredient in the *Roundup*^^[[1]](#footnote-1)^®^ family of agricultural herbicides. |
| **MON 87708** | A herbicide-tolerant soybean that produces a dicamba mono-oxygenase (DMO) protein from *Stenotrophomonas maltophilia* to confer tolerance to dicamba (3,6-dichloro-2-methoxybenzoic acid) herbicide. |
| **MON 87701** | An insect-protected soybean that produces the Cry1Ac insecticidal Cry protein derived from *Bacillus thuringiensis* to provide protection from feeding damage caused by targeted agricultural insect pests. |
| **MON 87751** | An insect-protected soybean that produces the Cry1A.105 and Cry2Ab2 insecticidal Cry proteins derived from *Bacillus thuringiensis* to provide protection from feeding damage caused by targeted agricultural insect pests. |
| **MON 87705** | A nutritionally-enhanced soybean with decreased level of saturated fats, increased level of oleic acid and associated decrease in linoleic acid, achieved through the use of *FATB1-A* and *FAD2-1A* endogenous gene segments. It also produces *5-enolpyruvylshikimate-3-phosphate synthase* protein from *Agrobacterium* sp*.* strain CP4 (CP4 EPSPS) to confer tolerance to glyphosate, the active ingredient in the *Roundup*^^[[2]](#footnote-2)^®^ family of agricultural herbicides. |
| **MON 87769** | A nutritionally-enhanced soybean which produces stearidonic acid (SDA), an omega-3 fatty acid, achieved through the introduction of genes encoding forthe production of Δ15 and Δ6 desaturases from *Neurospora crassa* and *Primula juliae*. |

*
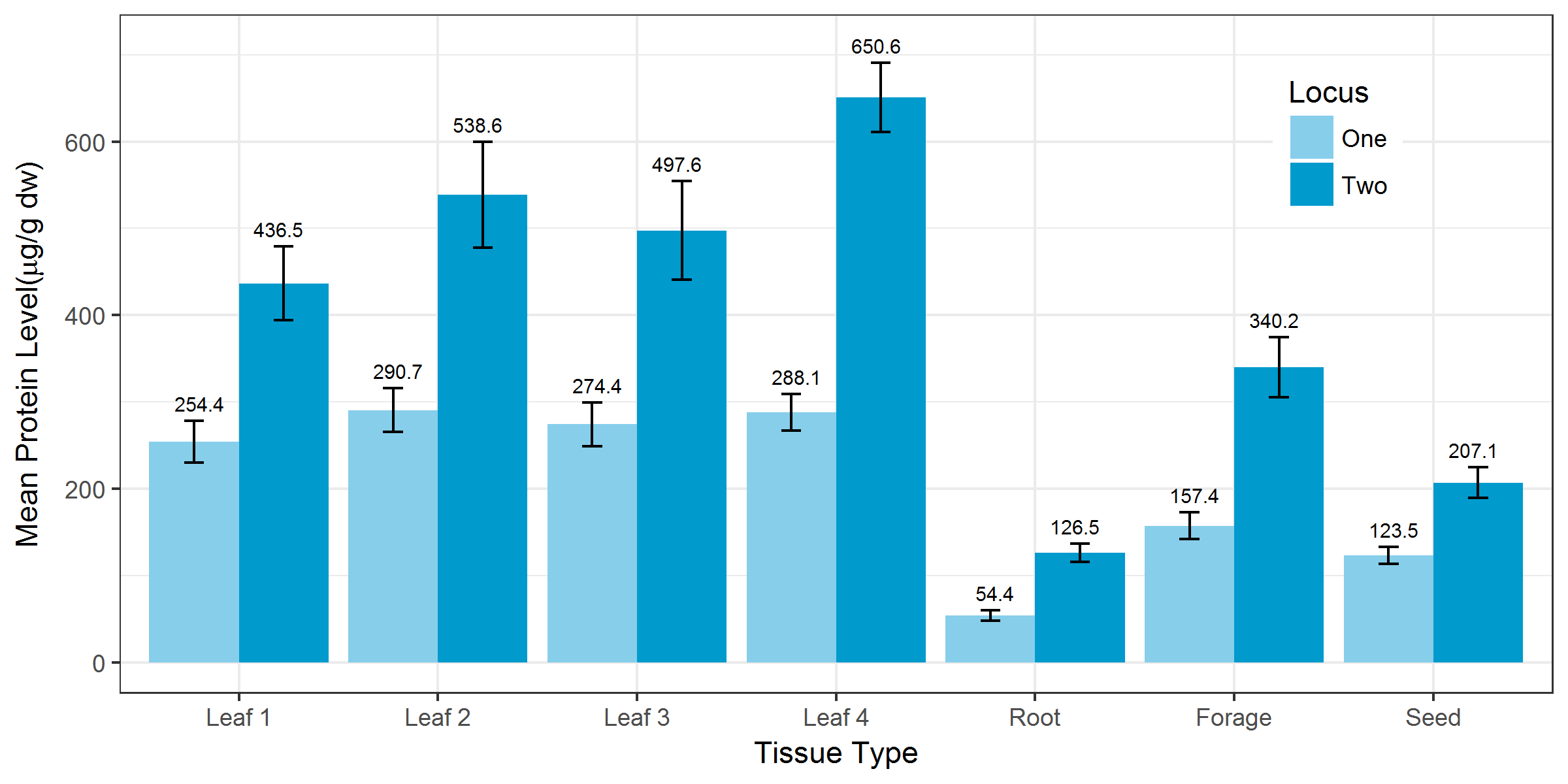
 S-Fig. 1*

*CP4 EPSPS expression (µg/g dw) by tissue types considering cp4 epsps locus number. Means were calculated across multiple studies representing multiple geographies, genetic backgrounds and stacked trait combinations. Entries with two cp4 epsps loci show significant difference in expression when compared to entries with one locus.*

1. ® *Roundup* is a registered trademark of Monsanto Technology LLC. [↑](#footnote-ref-1)
2. [↑](#footnote-ref-2)
